# Natural Bioactive Agents: Testable Stem Cell-Targeting Alternatives for Therapy-Resistant Breast Cancer

**DOI:** 10.3390/ijms26062529

**Published:** 2025-03-12

**Authors:** Nitin T. Telang

**Affiliations:** Cancer Prevention Research Program, Palindrome Liaisons Consultants, Montvale, NJ 07645-1559, USA; ntelang3@gmail.com

**Keywords:** cancer stem cells, drug resistance, natural products

## Abstract

Long-term treatment options for conventional chemo-endocrine therapy and molecular-pathway-based targeted therapy are associated with acquired therapy resistance and the emergence of drug-resistant cancer-initiating stem cell populations, leading to the progression of metastatic disease. These treatment options are based on the expression status of estrogen receptor-α (ER-α), progesterone receptor (PR) hormone receptors, and/or of human epidermal growth factor receptor-2 (HER-2). The breast cancer subtypes Luminal A, Luminal B, and HER-2-enriched express hormone/growth factor receptors and exhibit a favorable response to hormone receptor modulators and growth factor receptor antagonists. The triple-negative breast cancer subtype lacks the expression of hormone/growth factor receptors and responds only to cytotoxic conventional chemotherapy. The clinical limitations, due to the modest therapeutic responses of chemo-resistant cancer-initiating stem cells, emphasize the need for the identification of stem cells targeting testable alternatives for therapy-resistant breast cancer. Developed drug-resistant stem cell models exhibit upregulated expression of select cellular biomarker tumor spheroid (TS) formations and cluster of differentiation44 (CD44), DNA-binding protein (NANOG), and octamer-binding protein-4 (OCT-4) molecular biomarkers that represent novel experimentally modifiable quantitative endpoints. Naturally occurring dietary phytochemicals and nutritional herbs containing polyphenols, flavones, terpenes, saponins, lignans, and tannins have documented human consumption, lack systemic toxicity, lack phenotypic drug resistance, and exhibit preclinical efficacy. Constituent bioactive agents may provide testable stem cell-targeting alternatives. The present report provides an overview of (i) clinically relevant cellular models and drug-resistant cancer stem cell models for breast cancer subtypes, (ii) evidence for preclinical efficacy and mechanistic leads for natural phytochemicals and nutritional herbs, and (iii) the potential for the stem cell-targeting efficacy of natural bioactive agents as testable drug candidates for therapy-resistant breast cancer.

## 1. Introduction

Advanced-stage metastatic breast cancer represents a major cause of death in women. The American Cancer Society estimates 310,720 newly diagnosed breast cancer cases and 42,250 cancer-related deaths in women in 2025 [1].

Treatment options for breast cancer are dictated by the expression status of hormone and growth factor receptors. The treatment of choice includes endocrine therapy for hormone receptors expressing the Luminal A subtype. This therapeutic option uses selective estrogen receptor modulators such as tamoxifen and raloxifen, selective estrogen receptor degraders such as flauvestrant, and aromatase inhibitors such as letrozole, exemestane, and anastrozole. Human epidermal growth factor receptor-2 (HER-2)-targeted therapy, such as with the HER-2 selective antibodies trastuzumab and pertuzumab and the small-molecule receptor tyrosine kinase inhibitors lapatinib and neratinib, are in use for the Luminal B and HER-2-enriched subtypes. The triple-negative breast cancer (TNBC) subtype, because of the lack of hormone/growth factor receptor expression, responds only to conventional cytotoxic chemotherapy, which includes anthracycline, carboplatin, and paclitaxel [2].

The major limitations for clinical therapy of breast cancer are dependent on the specific molecular subtype. Thus, the Luminal A, Luminal B, and HER-2-enriched subtypes are traditionally treated with endocrine therapy and HER-2-targeted therapy, respectively. In contrast, the TNBC subtype is treated with cytotoxic chemotherapy. Clinical limitations due to long-term treatment include systemic toxicity, spontaneous and/or acquired phenotypic resistance, and the emergence of a therapy-resistant cancer-initiating stem cell population. Overall, these limitations favor the progression of stem cell-mediated metastatic disease, and therefore emphasize the need for the identification of these stem cells for targeting testable therapeutic alternatives.

Phenotypic resistance to breast cancer therapy is predominantly due to defective hormone receptor signaling, drug efflux, and resistance to molecular pathway selective small-molecule pharmacological inhibitors, leading to a putative drug-resistant stem cell population.

Natural products such as dietary phytochemicals, nutritional herbs, and their constituent bioactive agents have documented human consumption and evidence for growth-inhibitory efficacy in preclinical models. These natural products may represent testable alternatives as potential stem cell-targeting drug candidates for therapy-resistant breast cancer [3,4,5].

The present report provides an overview of (i) cellular models for breast cancer subtypes and for the drug-resistant cancer stem cell population, (ii) mechanistic leads for the growth-inhibitory efficacy of dietary phytochemicals and nutritional herbs, and (iii) a proof of concept for the applicability of cancer stem cell models to identify potential stem cell-targeting drug candidates for therapy-resistant breast cancer. This review provides a basis for future directions for clinically relevant basic research to identify novel therapeutic options.

## 2. Experimental Models

Cellular models for breast cancer and quantitative assays of cell proliferation and apoptosis remain as widely used experimental strategies for investigations focused on the anti-proliferative and pro-apoptotic effects of therapeutic agents. The data presented in Table 1 contain a short list of breast carcinoma-derived cell lines, the status of hormone and growth factor receptor expression, and representative cellular models for molecular subtypes of clinical breast cancer.

MCF-7 cells represent a model for the Luminal A breast cancer subtype. These cells express hormone receptors but lack the expression of the HER-2 receptor and exhibit a favorable response to several classes of hormone receptor modulators, receptor degraders, and aromatase inhibitors. The 184-B5/HER cell line lacks the expression of hormone receptors but expresses the HER-2 receptor. These tumorigenic cells exhibit overexpression of the HER-2 oncogene and represent a model for the HER-2-enriched breast cancer subtype. This subtype responds favorably to the HER-2-targeted antibodies trastuzumab and pertuzumab and to the small-molecule pan HER-2/EGFR receptor tyrosine kinase inhibitors lapatinib and neratinib. The MDA-MB-231 cell line lacks the expression of hormone and growth factor receptors and responds only to cytotoxic conventional chemotherapy including anthracyclines, carboplatins, and paclitaxel [2,6,7].

In contrast to non-tumorigenic mammary epithelial cell line 184-B5, the carcinoma-derived cellular models exhibit hyper-proliferation, downregulated apoptosis, accelerated cell cycle progression, and persistent anchorage-independent colony formation in vitro and tumor development in vivo [6]. The MCF-7, 184-B5/HER, and MDA-MB-231 cell lines represent relevant models for the Luminal A, HER-2-enriched, and triple-negative subtypes, respectively.

## 3. Natural Products as Test Agents

Unlike pharmacological therapeutic agents, natural products such as dietary phytochemicals and nutritional herbs and constitutive naturally occurring bioactive agents such as polyphenols, flavones, and terpenes may represent testable alternatives for breast cancer therapy. The natural products are notable for their anti-oxidant and anti-inflammatory properties and have documented preclinical growth-inhibitory efficacy on cellular models for different breast cancer subtypes. Select dietary phytochemicals and nutritional herbs, their origins, and their constituent major bioactive agents are described in Table 2.

In general, these natural products exhibit low systemic toxicity and have documented human consumption. These agents and/or their constituent bioactive agents may represent testable alternatives.

Dietary phytochemicals are represented by well-defined chemical entities that have distinct and unique mechanistic efficacy. Nutritional herbs are widely used in traditional Chinese medicine, and herbal formulations composed of multiple herbs are administered to patients as a decoction prepared in boiling water. To simulate patient consumption, non-fractionated aqueous extracts prepared from nutritional herbs have been used on cellular models for investigating their growth-inhibitory efficacy and identifying mechanistic leads. It is conceivable that multiple herbs may have multiple bioactive agents that function via synergistic interactions. The identification of major bioactive agents and their interactive mechanisms of action presents a challenge.

## 4. Mechanistic Leads for Growth-Inhibitory Efficacy

The mechanistic leads responsible for the growth-inhibitory effects of dietary phytochemicals and nutritional herbs are specific for individual cellular models.

### 4.1. Luminal A Model

In the Luminal A model exemplified by MCF-7 cells, oxidative metabolism of the common precursor estrone (E1) generates 2-hydroxyestrone (2-OHE1) via the C2-hydroxylation pathway and 16α-hydroxyestrone (16α-OHE1) via the C16α- hydroxylation pathway. Estrogen metabolites are commonly quantified either by using the tritium exchange assay that measures the formation of ^3^H_2_O in cells treated with stereo-specifically labelled estradiol, and the data are expressed as disintegrations per minute (dpm) per 10^6^ cells, or by using a gas chromatography–mass spectrometric assay that quantifies the formation of individual metabolites, and the data are expressed as ng/10^6^ cells. These estradiol metabolites exhibit distinct growth-modulatory effects. The 2-OHE1 metabolite has documented anti-proliferative effects, while the 16α-OHE1 metabolite has documented proliferative effects, as demonstrated in in vitro experiments with MCF-7 cells as well as in vivo experiments utilizing MCF-7 tumors developed from transplanted MCF-7 cells [8]. In these models, the test agents increase the formation of the anti-proliferative metabolite of estradiol. The 2-OHE1:16α-OHE1 ratio is commonly used as a quantitative endpoint. The nutritional herbs *Cornus officinalis* (CO), *Epimedium grandiflorum* (EG), and *Lycium barbarum* (LB) used as test agents in the MCF-7 model increase the ratio due to the increase in 2-OHE1 formation [9].

### 4.2. HER-2-Enriched Model

In the cellular model for the HER-2-enriched subtype exemplified by 184-B5/HER cells, the growth-inhibitory effects of the dietary phytochemicals used as test agents are included in Table 2. Treatment with these agents is associated with the inhibition of phosphorylation/activation of HER-2. An altered pHER-2:HER-2 ratio is indicative of the downregulation of HER-2 signaling. The pro-apoptotic effects of the test agents are associated with an increased incidence of the Sub G_0_ (apoptotic) cell population and an increase in the anti-apoptotic BCL-2 and pro-apoptotic BAX ratio [6,7]. For the quantitation of pHER-2, HER-2, and apoptosis-related BCL-2 and BAX proteins, cellular uptake of fluorescently labelled antibodies is monitored by flow cytometry, and the data are presented as relative fluorescent unit (RFU) per 10^6^ cells [6,7].

### 4.3. Triple Negative Model

In the cellular model for the TNBC subtype exemplified by MDA-MB-231 cells, the growth-inhibitory efficacy of nutritional herbs such as CO, *Dipsacus asperoides* (DA), and *Psoralia corylifolia* (PC) is associated with the inhibition of RB signaling via the cyclin D/CDK4/6/pRB axis [10,11]. Treatment with *Dryanaria fortunei* (DF) is associated with inhibition of the cyclin E/CDK2/pRB/E2F1 axis [12]. The expression patterns of relevant proteins for the signaling pathway represent quantitative endpoints. The pro-apoptotic effects of the test agents in this model are associated with an increased Sub G_0_ population and increased caspase 3/7 activity. The major mechanistic leads associated with the anti-proliferative and pro-apoptotic effects of the test agents are described in Table 3. The expression status of relevant proteins is monitored by Western blot assay and expressed as arbitrary scanning units (ASU). Caspase activity is expressed as relative luminescent units (RLUs) [12].

### 4.4. Drug-Resistant Cancer Stem Cell Models

Intrinsic resistance to conventional chemo-endocrine therapy and molecularly targeted pathway-selective therapy provides a rationale for developing models for clinically relevant cancer-initiating stem cells.

The normal stem cell population is essential during organogenesis and tissue regeneration and offers functional cellular homeostatic growth control via regulatory signaling pathways that include Wnt/β-catenin, Notch, and Hedgehog. The cancer stem cell population is characterized by disruption of these regulatory pathways [13]. Additionally, the tumor stem cell population exhibits activation of the RAS/RAF/MEK/ERK, PI3K, AKT, and mTOR survival signaling pathways that provide growth advantage to the cancer-initiating stem cell population [14,15]. The expression status of proteins is determined by Western blot assay, and the data are presented as the ratio between phosphorylated and total protein for MEK, ERK, PI3K, and AKT.

Optimized assays for the isolation of the putative stem cell population include the isolation of tumor spheroids, the sorting of cells positive for cell surface protein expression, such as CD44 and CD133, and the isolation of cells resistant to chemotherapeutics. The emergence of phenotypic resistance to chemotherapy suggests a strong clinical relevance and promise of translatability. Drug-resistant stem cell models for the Luminal A, HER-2-enriched, and TNBC breast cancer subtypes have been developed. Long-term treatment with a relevant chemotherapeutic agent at the respective maximum cytostatic concentration eliminates the drug-sensitive phenotype and promotes the growth of the resistant phenotype due to selective chemotherapeutic pressure. The resistant phenotypes are expanded under continued treatment, and the putative cancer stem cells are characterized. Thus, the selective estrogen receptor modulator tamoxifen (TAM) selects for resistant MCF-7 cells, the receptor tyrosine kinase inhibitor lapatinib (LAP) selects for resistant 184-B5/HER cells, and the chemotherapeutic anthracycline doxorubicin (DOX) selects for resistant MDA-MB-231 cells.

Drug-resistant stem models are characterized by the expression status of select biological and molecular markers. Tumor spheroids (TSs) are non-adherent colonies specific to the stem cell population and represent a biological marker. Cluster of differentiation44 (CD44) is a cell surface protein that represents a cellular marker for the stem cell population. The nuclear transcription factors DNA-binding transcription factor (NANOG) and octamer-binding protein-4 (OCT-4) represent molecular markers specific to the stem cell population. The tumor spheroid formation is quantified by the number of spheroid colonies. Preliminary evidence has suggested that phytochemicals such as curcumin (CUR), epigallocatechin gallate (EGCG), and carnosic acid (CA) inhibit TS formation. Cell surface marker CD44 and the transcription factors NANOG and OCT-4 are quantified by monitoring the cellular uptake of fluorescent antibodies. The primary data are presented as relative fluorescence units (RLUs) [7,16].

It is notable that the expressions of the OCT-4, KLF-4, SOX-2, c-Myc, and NANOG transcription factors are also documented to be specific for cancer stem cells [7,17] and are also essential for the maintenance of the induced pluripotent stem cell population [16,18]. Thus, biological, cellular, and molecular makers collectively provide quantitative endpoints for drug-resistant stem cell models. The biological, cellular, and molecular characterization of drug resistant stem cell models is presented in Table 4.

## 5. Experimental Modulation of Stem Cell Markers

The evidence documenting the modulation of stem cell markers by natural agents represents an important aspect supporting the feasibility of stem cells as models for the stem cell-targeting efficacy of test agents. For example, as natural products, all-trans retinoic acid (ATRA), an endogenous metabolite of vitamin A, and the naturally occurring rosemary terpene carnosol (CSOL) have exhibited inhibitory effects on inducible cyclooxygenase-2. This enzyme is induced by oncogenes and growth factors and is also upregulated in several organ-site cancers [19]. The inhibition of TSs and the downregulated expression of CD44, NANOG, and OCT-4 by ATRA and by CSOL provide a proof of concept supporting the stem cell-targeting efficacy of natural products (Table 5).

In conclusion, the evidence discussed in the present review relevant to the growth-inhibitory efficacy of dietary phytochemicals and herbal extracts on cellular models for breast cancer now provides a basis for extending investigations using xeno-transplant and/or ortho-transplant models. These investigations should provide confirmatory evidence where in vivo tumor formation represents the quantitative endpoint to facilitate analysis of the tumors to identify the growth-inhibitory mechanism of action. A schematic overview of the subject matter of the present review is presented in Supplementary Materials Table S1.

This schematic diagram summarizes the current clinical treatment options and predominant limitations, the established cellular models for select molecular subtypes of clinical breast cancer, the applicability of natural products and their advantages as testable alternatives, drug-resistant stem cell models, and stem cell-specific molecular markers. Notably, mechanistic biomarkers provide quantitative endpoints to investigate mechanisms of action and the identification of putative molecular targets for the efficacy of natural products.

## 6. Future Research Directions

The evidence discussed in the present review on stem cell models, the preclinical efficacy of dietary phytochemicals and nutritional herbs, and the proof of concept for stem cell-targeting efficacy together provide a scientifically robust rationale for future research directions that establish the clinical relevance of naturally occurring bioactive agents present in dietary phytochemicals and nutritional herbs as testable stem cell-targeting drug candidates for therapy-resistant breast cancer.

Network pharmacology, susceptible mechanistic pathways, and genomic-, proteomic-, and protein–protein-interaction-based functional assays are essential for the functional identification and analysis of the structure–function relationships of efficacious agents such as anti-proliferative hormone metabolites or bioactive agents present in nutritional herbal extracts on stem cell-targeting drug candidates for therapy-resistant breast cancer. This research direction involves the isolation/extraction/synthesis of the bioactive agents, functional assays for growth-inhibitory effects, omics-based functional mechanistic assays, and prioritization of efficacious agents for in vivo xeno-transplant or ortho-transplant assays. Mechanistic leads identified from network pharmacology and validated by functional assays have identified clinically relevant dietary phytochemicals such as CUR, EGCG, GEN, and RES and nutritional herbs from traditional Chinese medicine [3,4,5,20,21,22,23]. The network pharmacology-based experimental strategy should be useful for molecular subtypes of clinical breast cancer such as Luminal A, Luminal B, HER-2-enriched, and TNBC, where the gene expression status of genes coding for the hormone receptors ESR1 (ER-α), ESR2 (ER-β), and PR (progesterone receptor) and of genes coding for the growth factor receptors IGF1-R (insulin-like growth factor 1), EGFR (epidermal growth factor receptor), and HER-2 (human epidermal growth factor receptor-2) are associated with chemo-endocrine resistance. In addition, signaling pathways such as canonical or non-canonical ER signaling and tumor cell survival pathways such as RAS, PI3K, AKT, and mTOR may be susceptible to natural products. These lines of evidence provide a rationale for following future research directions.

### 6.1. ER-β Signaling

Estrogen receptor signaling has an opposing role in breast cancer progression. ER-α signaling functions as a positive growth regulator, while ER-β signaling functions as a negative growth regulator in estrogen-responsive breast cancer [24]. Down-modulation of ER-β signaling is associated with endocrine resistance via the MAPK and GCPR pathways [25]. Naturally occurring phytoestrogens present in nutritional herbs influence the binding of estrogen response elements and the expression of ER-β target genes [26,27]. Major bioactive agents present in nutritional herbs, including flavones, lignans, and saponins, functioning as ER-β agonists, may represent testable drug candidates. The expression status of ER-β at the protein level is quantified by Western blot assays, and quantitation at the gene expression level is provided by RT-PCR assays.

### 6.2. Telomerase Expression

Breast carcinoma-derived immortalized cancer-initiating stem cells universally express human telomerase reverse transcriptase (hTERT). This enzyme is responsible for adding 5′ TTAGGG 3″ repeat nucleotide sequences to chromosomal telomeres and confers persistent replicative potential to the cancer stem cells [28,29]. In the response of breast cancer to therapy, telomerase expression is responsible for protection against therapy by promoting therapeutic resistance [30]. Thus, hTERT may represent an attractive cancer therapeutic target [31,32]. The selective inhibitory efficacy of several pharmacological agents such as nucleoside analogs, small-molecule inhibitors, and anti-sense molecules have been documented to be effective for the inhibition of hTERT [33]. However, the clinical use of these pharmacological agents is challenging, predominantly because of detectable off-target effects and systemic toxicity. In this context, naturally occurring anti-proliferative hormonal metabolites and herbal bioactive agents may function as novel stem cell-targeting telomerase inhibitors. Conventionally, the telomeric repeat amplification protocol (TRAP) assay is used as a quantitative endpoint [28].

### 6.3. Epigenetic Modifiers

Epigenetic modifications impact cell plasticity and gene transcriptional activity. Nuclear histone modifications, DNA methyl transferase activity, and gene promoter methylation are essential for epigenetic functioning, and mechanistically, methyl donors generated via one-carbon metabolism play an important role in this process [34]. In particular, histone acetylation DNA-modifying enzymes and CpG island modifications play important functions as epigenetic modifiers in cancer metastasis [35]. In addition, naturally occurring dietary molecules have documented effects on DNA methylation, histone acetylation, and epigenetic gene regulation [36]. Thus, naturally occurring bioactive agents functioning as epigenetic modifiers may be effective in therapy-resistant breast cancer stem cells. Specific quantitative assays for histone modification, DNA methylation, and CpG island modification are available [34,35].

### 6.4. Epithelial–Mesenchymal Transition (EMT)

This process characterizes cellular plasticity in cancer stem cells and is associated with reciprocal modulation in epithelial–mesenchymal transition (EMT) and mesenchymal–epithelial transition (MET). During EMT, epithelial markers are upregulated and mesenchymal markers are downregulated. This expression pattern is reversed during MET. The reciprocal transition has essential roles in the modulation of cellular migration and invasion. Specific cellular proteins, cadherin and vimentin, and the transcription factors SLUG, SNAIL, and ZEB represent quantitative endpoints [36]. Additionally, the NFkB-STAT-3 signaling pathway plays critical roles in EMT/MET signaling pathways [37]. Long-coding RNA and microRNA have been used to investigate cellular motility, stemness, and drug resistance [38]. In addition, transforming growth factor-β (TGF-β) expression has been associated with cancer stem cells and cellular heterogeneity [39,40]. The anti-cancer potential of phytochemicals has been associated with the regulation of EMT [41]. Naturally occurring bioactive agents that are effective via these pathways may represent testable drug candidates. EMT markers such as E-cadherin and vimentin are commonly quantified by immuno-fluorescent assays. Nuclear transcription factors such as SLUG, SNAIL, and ZEB are quantified by Western blot assays at the protein level and by RT-PCR assays at the gene expression level [35,36].

Preclinical data from investigations using established cells lines and developed drug-resistant stem cell models [7,14,22,42] are dependent on extrapolation for their clinical relevance and translatability. This limitation may be reduced by developing stem cell models from therapy-resistant patient-derived tumor explant [43] and tumor organoid samples [44,45]. A recent comparative analysis of cell-derived tumor explant (CDTX) and patient-derived tumor explant (PDTX) models has provided evidence that ortho-transplants from CDTX or PDTX represent preferred techniques for testing bioactive agents as potential new drug candidates [46].

## Figures and Tables

**Table 1:** Breast cancer subtypes.

Cell Line	Receptor Status			Preclinical Model
	ER-α	PR	HER-2	
MCF-7	+	+	−	Luminal A
T47D	+	+	−	Luminal A
BT474	+	+	+	Luminal B
MDA-MB-361	+	+	+	Luminal B
SKBr-3	−	−	+	HER-2-enriched
184-B5/HER	−	−	+	HER-2-enriched
MDA-MB-231	−	−	−	Triple negative

ER-α, estrogen receptor-α; PR, progesterone receptor; HER-2, human epidermal growth factor receptor-2; +, positive; −, negative.

**Table 2:** Naturally occurring test agents.

Test Agent	Origin/Source	Bioactive Agent
Dietary Phytochemical		
CA	Rosemary leaf/stem	Terpene
CSOL	Rosemary leaf/stem	Terpene
EGCG	Green tea	Polyphenol
GEN	Soy	Flavone
Nutritional Herb		
CO	Fruit	Anthocyanin
EG	Leaf/stem	Prenylflavone Icariin, Icaritin
DA	Root	Triterpene
LB	Fruit/bark	Flavone, tannin
PC	Seed	Terpene

CA, carnosic acid; CSOL, canosol; EGCG, epigallocatechin gallate; GEN, genistein; CO, *Cornus officinalis*; EG, *Epimedium grandiflorum*; DA, *Dipsacus asperoides*; LB, *Lycium barbarum*; PC, *Psoralia corylifolia*.

**Table 3:** Mechanistic leads.

Cell Line	Mechanistic Lead	Endpoint
MCF-7	Estradiol metabolism	2-OHE1: 16α-OHE1
184-B5/HER	HER-2 signaling	pHER-2: HER-2
	Apoptosis	Sub G0, BCL-2, and BAX
MDA-MB-231	RB signaling	pRB: RB, Cyclin D1, CDK4/6, pRB, Cyclin E, CDK2, pRB, E2F1
	Apoptosis	Sub G0, caspase 3/7, Z-VAD-FMK, and PARP-1

2-OHE1, 2-hydroxyestrone; 16α-OHE1, 16α-hydroxyestrone; pHER-2, phosphorylated human epidermal growth factor receptor-2; HER-2, human epidermal growth factor receptor-2; SubG0, apoptotic phase of the cell cycle; BCL-2, B-cell lymphoma protein-2; BAX, B-cell associated protein X; pRB, phosphorylated retinoblastoma; CDK, cyclin dependent kinase; E2F1, E2f family transcription factor; Z-VAD-FMK, anti-caspase peptide; PARP-1, poly(ADP-ribose) polymerase-1 (summarized from [10,11,12]).

**Table 4:** Stem cell models.

Drug Resistance	Stem Cell Marker Expression ^a^			
	TS	CD44	NANOG	OCT-4
TAM-R	2.0X	4.7X	2.8X	3.3X
LAP-R	2.5X	4.2X	4.9X	2.0X
DOX-R	3.1X	4.9X	1.9X	2.4X

^a^ Relative to expression in drug-sensitive phenotype. TS, tumor spheroid; CD44, cluster of differentiation44; NANOG, DNA-binding transcription factor; OCT-4, octamer-binding protein-4; TAM-R, tamoxifen resistant; LAP-R, lapatinib resistant; DOX-R, doxorubicin resistant (summarized from [7,18]).

**Table 5:** Modulation of stem cell marker expression in LAP-R stem cells.

Treatment	Concentration (IC 90, µM)	Stem Cell Marker Expression (Inhibition % Control) ^a^			
		TS	CD44	NANOG	OCT-4
ATRA	3	69	81	82	72
CSOL	5	83	86	78	78

^a^ Relative to solvent control. TS, tumor spheroids; CD44, cluster of differentiation44; NANOG, DNA-binding nuclear transcription factor; OCT-4, octamer-binding prtoeein-4; ATRA, all-trans retinoic acid; CSOL, carnosol (summarized from [6]).

## Data Availability

The data sets used in this review are available from the author on reasonable request.

## Supplementary Materials

The following supporting information can be downloaded at: https://www.mdpi.com/article/10.3390/ijms26062529/s1.
