# Supplementary material for: Natural Bioactive Agents: Testable Stem Cell-Targeting Alternatives for Therapy-Resistant Breast Cancer
Source: Int J Mol Sci. 2025 Mar 12;26(6):2529. doi: 10.3390/ijms26062529 (PMC11942498; doi:10.3390/ijms26062529)
Supplement: Supplementary file 1 [file ijms-26-02529-s001.zip › ijms-3461770-supplementary.pdf]

Table S1. Natural Products: Testable Alternatives for Breast Cancer.

Treatment Option	Breast Cancer Model	Mechanistic Biomarker
Endocrine therapy	Luminal A	ER, E2 metabolite
Endocrine, HER-2 therapy	Luminal B	ER, E2 metabolite
HER-2 therapy	HER-2-enriched	HER-2 signaling
Chemotherapy	TNBC	Cell cycle, apoptosis
**Limitation:**		
Phenotypic resistance		
Drug resistant cancer stem cells		
**Natural Products**		
**Dietary phytochemicals**	Luminal A	ER, E2 metabolite
Polyphenols, Flavones	HER-2-enriched	Cyclin D1, Cyclin B,
Terpenes		HER-2, Bcl-2, BAX
**Chinese nutritional**	Luminal A	E2 metabolite
**Herbs**	TNBC	Cyclin D1, Cyclin E,
		CDK, RB,
		Bcl-2, BAX,
		Caspase, PARP1
**Advantage:** Low toxicity, Human consumption		
**Drug resistant cancer stem cells**		
	TAM-R	CD44, NANOG, OCT-4
	LAP-R	
	DOX-R	
**Advantage:** Stem cell targeting		

ER, estrogen receptor; E2, estradiol; HER-2, human epidermal growth factor receptor-2; Bcl-2, B cell lymphoma protein-2; BAX, B cell associated protein x; CDK, cyclin dependent kinase; RB, retinoblastoma; PARP-1, poly (ADP-ribose) polymerase-1; TNBC, triple negative breast cancer; TAM-R, tamoxifen resistant; LAP-R, lapatinib resistant; DOX-R, doxorubicin resistant; CD44, Cluster of differentiatiuon44; NANOG, DNA-binding nuclear transcription factor; OCT-4, octamer-binding prtein4.
